# Proposal of a Theoretically Feasible Method to Perform Perilymph Sampling in Clinical Settings

**DOI:** 10.3390/life14101323

**Published:** 2024-10-18

**Authors:** Federico Maria Gioacchini, Massimo Re, Alfonso Scarpa, Giuseppe Chiarella, Pasquale Viola, Davide Pisani, Giannicola Iannella, Massimo Ralli, Arianna Di Stadio

**Affiliations:** 1 Ear, Nose, and Throat Unit, Department of Clinical and Molecular Sciences, Polytechnic University of Marche, Via Conca 71, 60020 Ancona, Italy; remassimo@gmail.com; 2 Department of Medicine and Surgery, University of Salerno, 84084 Salerno, Italy; alfonsoscarpa@yahoo.it; 3 Unit of Audiology, Regional Centre of Cochlear Implants and ENT Diseases, Department of Experimental and Clinical Medicine, Magna Graecia University, 88100 Catanzaro, Italy; chiarella@unicz.it (G.C.); pasqualeviola@unicz.it (P.V.); davidepisani@unicz.it (D.P.); 4 Department of Organi di Senso, Sapienza University, 00185 Rome, Italy; giannicola.iannella@uniroma1.it (G.I.); massimo.ralli@uniroma1.it (M.R.); 5 GF Ingrassia Department, Otolaryngology, University of Catania, 95124 Catania, Italy

**Keywords:** perilymph, clinical diagnosis, endoscopy, inner ear disease

## Abstract

The ability to analyze perilymph could allow inner ear pathologies to be studied. However, today, perilymph sampling is only performed for research purposes because of the risk of negative outcomes such as hearing loss or balance disorders. This paper aims to analyze the current literature on perilymph sampling and propose a method to collect perilymph in clinical settings. The published literature on perilymph sampling and its analyses was screened, and the results were analyzed and discussed in this mini review. Also, articles that discussed microneedle technology were reviewed and included in the analysis of the data. Based on the results of this review, we would like to propose a feasible technique to perform perilymph sampling in clinical settings. A total of eight studies analyzing perilymph were identified; data on proteomic, metabolomic and miRNA features present within human perilymph were collected and described. Two articles describing the use and auditory outcomes post microneedle drug injection into the inner ear were identified. Based on the methods for perilymph sampling described in humans and the recent innovations introduced by the use of microneedles, we suggested a feasible method to collect perilymph in the outpatient setting. The analysis of perilymph undoubtedly represents a valid instrument to fully understand inner ear diseases. A combination of traditional and innovative techniques, such as gaining access to the round window through the transcanalar approach using micro-endoscopes and microneedles to perform sampling, might simplify the sampling procedure and make it practicable in a clinical setting.

## 1. Introduction

Inner ear disorders represent the most frequent sensory diseases worldwide; these include deafness, sensorineural hearing loss, Meniere’s disease, benign paroxysmal positional vertigo, labyrinthitis, secondary endolymphatic hydrops and perilymphatic fistula [1]. The cochlea—the auditory organ—and the vestibular system—the equilibrium organ—compose the inner ear, a small structure located in the temporal bone. The inner ear has an external bony shell, the *bony labyrinth*, and an internal soft component, the *membranous labyrinth*. The interspace between the *bony labyrinth* and *membranous labyrinth* is filled with perilymph, a standard Na+-based extracellular solution. Endolymph, a unique extracellular solution characterized by high K+ and low Ca2+ and Na+ concentrations, circulates into the *membranous labyrinth*. The helicotrema connects *scala tympani* and *scala vestibuli* from the bottom the to the apex of the cochlea; the two scales contain perilymph. The *scala media*, or the middle chamber, contains endolymph. This structure is connected to the vestibular system through *the ductus reuniens* [2]. The *scala tympani*, which is close to the round window membrane (RWM), is connected to the cochlear aqueduct; the latter is directly connected to the subarachnoid space that contains cerebrospinal fluid (CSF). CSF is one of the possible routes for perilymph refueling; however, close to the other perilymphatic capillaries, there are areas that can produce perilymph. 

The *bony labyrinth* is the densest and hardest bone of the human body; therefore, access to its contents requires a surgical approach (an invasive procedure), making it challenging for diagnostic purposes [3]. Magnetic Resonance Imaging (MRI) has been used to analyze the characteristics of endolymphatic hydrops [4,5]; however, details about the pathological changes in the *membranose labyrinth* were hard to detect. In fact, MRI can only focus on the structural characteristics of the endolymphatic hydrops in Meniere’s disease. However, in this condition, the content of perilymph is absolutely an important aspect, so perilymph sampling can support correct diagnosis and adequate treatments. 

The inner ear is a highly compartmentalized microenvironment, and these compartments are connected to the central nervous system. The endolymphatic compartment, inside the *membranous labyrinth,* certainly represents a more complex area of the human inner ear. Various pathologies affect its cellular structures, causing a series of audio-vestibular symptoms. The violation of the tight junction system, which separates the *scala media* (endolymph compartment) from the remainder of the inner ear, causes complete hearing loss; this represents the major limitation of inner ear biopsies and content sampling [6]. In any case, since the perilymphatic and endolymphatic compartments are connected, the analysis of perilymph could clarify what happens both in the cochlea and in the vestibular apparatus. The perilymph contents (ions, proteins) can vary between patients and healthy subjects. This information could help to create tailor-made treatment in line with the current idea of precision medicine; therefore, the analysis of perilymph could be extremely relevant in clinical settings. 

Today, the perilymphatic route has been widely investigated for the delivery of drugs to the inner ear, because using this approach makes it possible to avoid systemic high-dose treatments (generally used to treat inner ear diseases) and their related risks, i.e., steroids [7]. It is becoming common opinion that the perilymph approach could support both diagnostic and therapeutic aims. In fact, the technical limitations of the methodologies currently used to diagnose inner ear pathologies do not allow clear and well-defined diagnoses of audio-vestibular disorders, limiting “ad hoc” therapeutic solutions. 

The identification of a feasible and easy technique to collect perilymph could allow for the accurate analysis of inner ear fluid for electrochemical, genetic, or proteomic features.

Soon, perilymph sampling might be the best tool to identity and treat inner ear problems, which manifest with audio and vestibular symptoms. 

This review aims to discuss a theoretical method to perform perilymph collection and to explain how to do it in a clinical setting using the currently available otolaryngology technologies.

## 2. The Potential Bridging Role of Perilymph in Future Audiological Practice

Table 1 summarizes studies analyzing perilymph components in human samples. Even though the literature on this topic is still growing, perilymph sampling seems to be very promising.

## 3. Perilymph Sampling in Actual Ontological Surgery and Possible Evolution Using Microneedles

Perilymph sampling, when necessary, is generally performed via the RWM during stapedectomy, labyrinthectomy and cochlear implantation [18]. 

Sterile glass capillary tubes are commonly used to perform intraoperative sampling by the RWM; the amount of extracted perilymph must be as minimal as possible to preserve hearing [1]. In addition, the volume of the sample cannot be uniform due to the variability related to the operator skills and the caliber of the chosen cannula. Moreover, puncture of the RWM by a glass capillary tube may cause CSF leakage into the scala tympani with contamination of the sample [6]. 

To date, only a few people are experts in this technique and can perform perilymph sampling with minimal risk to the patient’s auditory functions (i.e., deafness). In fact, it is known that an excessive loss of perilymph during stapedectomy does not allow the patients to obtain satisfactory auditory results [19]. 

Recently, microneedles have been developed to inject drugs and genetic material into the inner ear [20,21]. This technology has been created to minimize the trauma and to facilitate the recovery of hair cells when an inner ear injection treatment is performed. The method allows access to the inner ear with minimal anatomic and functional damage. 

Microneedles also allow fluids to be aspirated from the cochlea with more precision than tubes and with the minimal extraction of perilymph in an atraumatic manner [1]. 

Additionally, microneedles can be 3D-printed, reducing the cost of the production and facilitating the availability of this technology in many centers [20]. The technique is safe, as shown by the performance of two perforations and aspirations in eight guinea pigs to extract cochlin. The protein was identified in all samples, and the hearing tests performed in the animals showed only mild hearing loss at 1–4 kHz and 28 kHz, mostly consistent with conductive hearing loss. The analysis of the membrane using a confocal microscope showed complete healing of the RWM holes [20]. 

Microneedles are available in different materials [6,20,21]; however, for our proposed technique, we suggest that a stainless steel microneedle might be the best option to combine flexibility and resistance [6,21].

## 4. Proposal for an Ideal Surgical Approach to Perform Routine Perilymph Sampling in Humans

To perform perilymph sampling without bone removal and with minimum trauma by a natural route, the only access is through the RWM. This structure has a surface of 2.3 mm^2^ [22] with 70 μm thickness [23] and is very close to the basilar membrane—approximately 1.2 mm [24]. 

Peter et al. [6] proposed the traditional microscopic transcanal approach through the tympanomeatal flap to reveal the structures of the middle ear. The round window niche would be then identified, and its bony overhang removed.

Peter’s approach presents some limitations; first, since it is usually performed under local anesthesia, the patient’s cooperation is needed and is not always possible. In addition, the tympanomeatal flap needs time to be correctly and safety performed. Finally, despite being minimal, the risk of external auditory canal (EAC) bleeding is possible due to the high vascularization of the area. In our opinion, the procedure must be carried out with minimal trauma to the ear and minimal distress for patients, so access to the RWM by traditional surgery is not feasible, especially as a diagnostic tool. 

In fact, in those patients who do not need surgery, an approach to the RWM through the EAC—transcanalar approach—with minimal traumatism to the tympanic membrane might be an interesting method. Because of the anatomical position of the RWM (median angle of 113°), the approach to this structure could be challenging without the access to the middle ear. The only access seems to be by elevating the tympanomeatal flap, but its feasibility is limited for the abovementioned reasons.

In any case, today, there are new technologies that, combined together, could simplify perilymph sampling and reduce the risk related to the procedure. Endoscopy is one of the technologies that might be used in this scope.

An endoscope allows for the direct visualization of the ear, which is the reason why it has been used in middle [25] and inner ear surgery [26]. A rigid endoscope has high magnification with different angle views (45°or 70°) that help the user to see “behind the corner”. In 2014, Marchioni et al. [27] described six patients who underwent endoscope-assisted cochlear implantation surgery; the endoscope allowed the electrode to be placed in the round window without mastoidectomy needing to be performed. An endoscope (0°, 3 mm wide, 15 cm in length) coupled to a high-definition (HD) camera was inserted through the transcanal surgical route created by standard post-auricular incision. During the following years, additional advances in this original technique [27] were proposed by other researchers. 

Gülşen et al. [28], in a clinical report, described a fully endoscopic transcanal revision cochlear implant. However, despite the excellent result achieved, Gülşen performed the tympanomeatal flap before the introduction of the rigid endoscope into the middle ear cavity, making his described technique a non-fully endoscopic surgery and adding an invasive passage, the tympanomeatal flap, exactly like Marchioni et al. [27].

In our opinion, although the angle of the RWM may be variable, as well as the RW niche, the use of an angled micro-endoscope that allows the full inspection of the posterior middle ear portion could overcome the limitation of the invasive (despite being minimally so) tympanomeatal flap approach.

We think that two options could be considered using the combination of an endoscope and microneedle. One option could be using a single flexible device comprising a microneedle and an HD camera on the tip that gives to access to the RWM by the creation of a small hole on the tympanic membrane (smaller than tympanocentesis), using one single hand only. This “one-hand” option needs a flexible and strong-enough material to support the camera on its tip and to maintain the needed flexibility to be oriented and inserted in the RWM.

Despite being intriguing, the single-hand technique currently has poor feasibility because the needed technologies are not available yet. 

**Figure 1:**
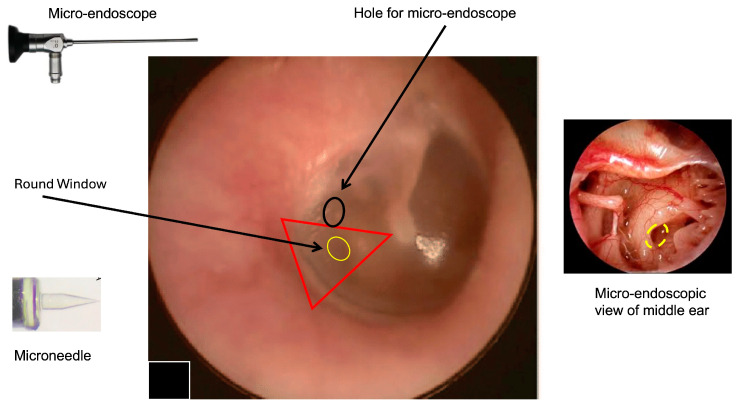
Right tympanic membrane (TM). The image shows the hypothetical area that can be used to access to the round window using the transcanal approach; one hole is for the endoscope (black circle); the yellow area represents the round window where the microneedle will be introduced. The right triangle represent the area where it is possible to identify the round window. The two points of access will be in the posterior quadrants of TM (pars tensa). To perform the procedure, the patient is supine. Local anesthesia with lidocaine spray will be performed. After performing a small incision on the tympanic membrane (black ring), a 0° endoscope is inserted to visualize the middle ear and the position of the round window (interrupted yellow ring) before performing the perilymph sampling by microneedle. This approach will allow a direct visualization of the sampling area and allow eventual perilymph leakage at the end of the procedure to be monitored. Other degree (30°, 45°, 70°) endoscopes could be considered the basis of the specific anatomical conditions and the personal experience of the surgeon (the image of the TM was modified from Di Stadio et al., Brain Sci, 2024 [8] to introduce the location of the additional hole for micro-endoscope insertion).

At the present time, a “two-hand” option could be the most achievable option. The two-hand technique might be performed by inserting a rigid micro-endoscope (handled by the left hand) through the tympanic membrane and a flexible device with a microneedle on the tip (using the right hand) to perform perilymph sampling. Because a single hole could not allow a micro-maneuver to be performed without the risk of severe damage to the tympanic membrane, we propose a double hole technique (as for all types of endoscopic surgeries) with a hole for the micro-endoscope and a second hole (smaller than the first) to introduce the sampling device (Figure 1). We think that a micro-endoscope that is 1 mm wide could allow sufficient visualization of the RWM and good illumination of the middle ear cavity, leaving enough space for maneuver even in patients with small external auditory canals. 

Thanks to development of these technologies, both micro-endoscopes and microneedles might be easily available in the future.

The main limitation of this technique is the learning curve; however, because of the reduction in the risk and the easiness of performing a view procedure, the training should not be a problem for a physician interested in this approach, especially considering the benefit achievable by its use in perilymph sampling.

It is important to underline that this is only a theoretical proposal. Studies on cadaveric samples must be performed to verify the feasibility of our proposed technique. Moreover, these studies could also evidence the difficulties in perilymph sampling based on the different anatomy of the RWM.

## 5. Conclusions

Developing technologies can be an interesting opportunity to facilitate perilymph sampling so as to use it in clinical settings. The combination of a micro-endoscope and a flexible device for perilymph sampling might simplify the procedure, rendering it possible to be performed in clinic to carry out the precise diagnoses of inner ear diseases with adequate tailor-made treatment (the principle of personal medicine). 

In the future, robotic development and the use of Artificial Intelligence (AI) might further improve and simplify the extraction of perilymph in a totally atraumatic way. In fact, thanks to AI, it would be possible to identify the best pressure to apply and then move the robotic arm based on AI suggestions. The robotic arm can perform extremely precise movements that are a fundamental aspect in a such small area like the EAC. The combination of AI and robotic surgery could minimize all the risk related to human operators, like small hand tremors or the excessive use of pressure when creating an RWM hole. 

## Figures and Tables

**Table 1:** Published studies on analysis of human perilymph components (modified from Di Stadio et al., Brain Sci, 2024 [8]).

	Years	Total Number of Participants	Cause of Hearing Loss	Control Group Without SNHL	Surgical Procedure/Sampling Tool	Perilympatic Components Analyzed	Main Findings
Mavel et al. [9]	2018	23	CMV; trauma; MD	None	CI/ needle 22 g	Metabolome	Fingerprinting was obtained from 98 robust metabolites.
Rasmussen et al. [10]	2018	16	VS	None	TC, L/ n.a.	Proteome	Alpha-2-HS-glycoprotein, P02765, was shown to be an independent variable for tumor-associated hearing loss.
Lin et al. [11]	2019	5	MD	Yes	TO, TC, L needle 28 g	Proteome	A total of 228 common proteins were identified in the perilymph of patients with Meniere’s disease, 38 of which were significantly differential in abundance.
Thrin et al. [12]	2019	19	n.a.	None	CI/ needle 22 g	Metabolome	A total of 106 different metabolites were identified. Metabolomic profiles were significantly different in subjects with ≤12 or >12 years of hearing loss
de Vires et al. [13]	2019	38	MD, CMV, EVA, CHARGE, meningitis	Yes	CI/ microglass capillary	Proteome	(1) BDNF was found to be expressed in cochlear tissue in individuals with normal hearing; (2) there was an overall decreased level of expression of BDNF-regulated proteins in profoundly hearing-impaired patients compared to patients with some residual hearing.
Warnecke et al. [14]	2019	43	n.a.	None	CI/microglass capillary	Proteome	Multiplex proteins were identified in very small samples (1 microL or less). Higher IGFBP1 levels were identified in deaf patients compared to patients with residual hearing.
Shew et al. [15]	2021	10	MD	Yes	S, L/microglass capillary	miRNA	Sixteen differentially expressed miRNAs were identified in the perilymph of patients with MD.
Schmitt et al. [16]	2021	31	MD, OS, EVA	None	CI/ n.a.	Proteome	Overall, 895 different proteins were found in all samples. Based on quantification values, a disease-specific protein distribution in perilymph was demonstrated.
van Dieken et al. [17]	2022	38	n.a.	None	CI/microglass capillary	Proteome	The authors propose a human atlas of protein of the cochlea.

OS: otosclerosis; VS: vestibular schwannoma; CMV: cytomegalovirus infection; g: gauge; MD: Meniere’s disease; EVA: enlarged vestibular aqueduct; CI: cochlear implantation; TO: transotic approach; TC: transcochlear approach; S: stapedectomy; L: labyrinthectomy; IGFB: insulin-like growth factor protein; BDNF: brain-derived neurotrophic factor; CHARGE: coloboma, heart defects, choanal atresia, retarded growth and development, genital hypoplasia, ear anomalies.

## Data Availability

Data available upon request to the corresponding author.
